# Plasmablastic Lymphoma Mimicking Osteomyelitis of Femur in an Immunocompetent Individual: A Case Report

**DOI:** 10.7759/cureus.21183

**Published:** 2022-01-12

**Authors:** Sheza Malik, Abdullah Zaki, Muhammad Usman Shabbir, Taimoor Hussain

**Affiliations:** 1 Medicine, Army Medical College, Rawalpindi, PAK; 2 Internal Medicine, Shifa International Hospital, Islamabad, PAK; 3 Medicine, Shifa International Hospital, Islamabad, PAK; 4 Neurology, Bolan Medical College, Quetta, PAK

**Keywords:** plasmablastic lymphoma, immunocompetent, case report, osteomyelitis, thigh muscles, dynamic compression plates, mimicking abscess

## Abstract

Plasmablastic lymphoma (PBL) is an aggressive type of diffuse large B-cell lymphoma. It is most commonly seen in patients with human immunodeficiency virus (HIV) infection and other immunodeficiencies manifesting commonly in the form of oral lesions.

Here, we report a case of an HIV-negative, immunocompetent elderly male who presented with a painful solitary tender lesion on the right anterior lateral thigh. A preliminary diagnosis of osteomyelitis (right femur) from a possibly infected dynamic compression plate was made following initial ultrasound and MRI of the right lower extremity. An attempt was made to incise and drain the lesion, which was abruptly stopped as it resulted in drainage of copious blood, leading to hemodynamic instability. Histopathology of the specimen revealed findings consistent with PBL. The diagnosis of PBL was further confirmed by immunohistochemical staining, which was positive for CD138, MUMI, and CD56 and negative for CD20 and ALK. Due to its rarity and heterogeneous presentations, PBL could be easily overlooked clinically in immunocompetent patients. Therefore, our case highlights the importance of considering the diagnosis of PBL even in lesions whose course is consistent with other infectious bone pathologies.

## Introduction

Plasmablastic lymphoma (PBL), according to the World Health Organization (WHO) classification 2016, is characterized by diffuse proliferation of large neoplastic cells morphologically resembling B immunoblasts with the immunophenotype of plasma cells [1]. Patients suffering from PBL frequently have an underlying immunocompromised state such as HIV infection [2,3]. Due to its strong association with HIV, the diagnosis of PBL should always raise suspicion about undiagnosed HIV infection [4,5].

While HIV-associated PBL accounts for most PBL diagnoses, there are increasingly reported cases in an otherwise healthy population. Most commonly, it presents with mucosal oral lesions [4]. However, it has also been reported to involve breast [6], ovary [7], gastrointestinal tract [8], skin [9], and bones [10]. Herein, we report a case of PBL involving anterior thigh muscles quadriceps femoris in an immunocompetent, non-HIV-infected elderly male. To our knowledge, there are only two more reported cases of PBL with soft tissues involvement in an immunocompetent patient [11,12].

## Case presentation

A 58-year-old male patient presented to the surgical outpatient clinic with undocumented high-grade fever and painful right thigh swelling for one month. He has a history of diet-controlled diabetes mellitus and a motor vehicle collision (MVC) in 2003 and open reduction and internal fixation of the right femur. According to the patient, he was in his usual state of health until one month ago when the swelling, which now involves almost one-fifth of the thigh, started growing rapidly. He also reported associated high-grade fevers and persistent sharp, non-radiating thigh pain, which would aggravate with ambulation.

**Figure 1:**
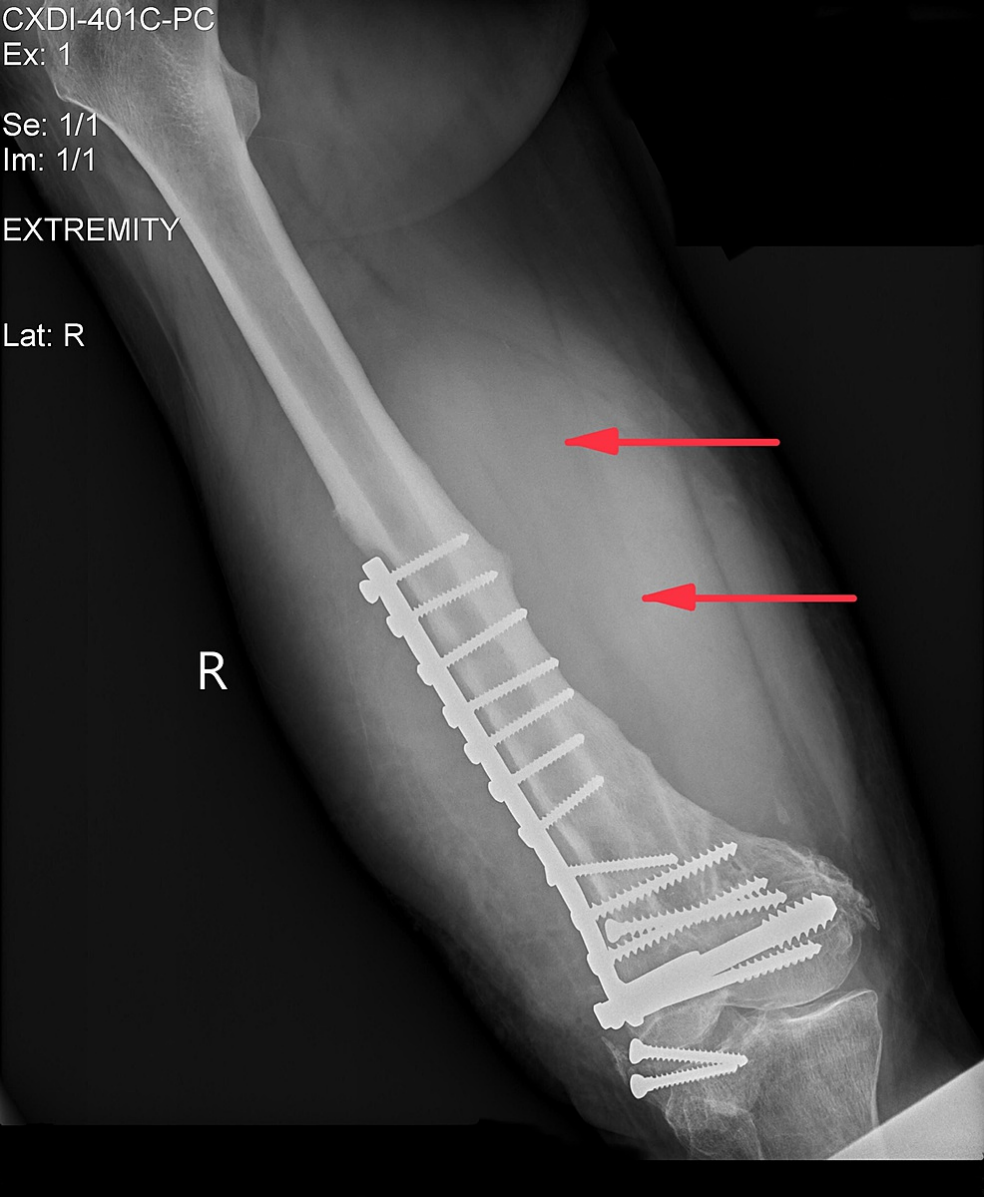
X-ray of the right femur (AP view) showing DCP and surrounding hyperlucency. AP - anteroposterior,  DCP - dynamic compression plate

**Figure 2:**
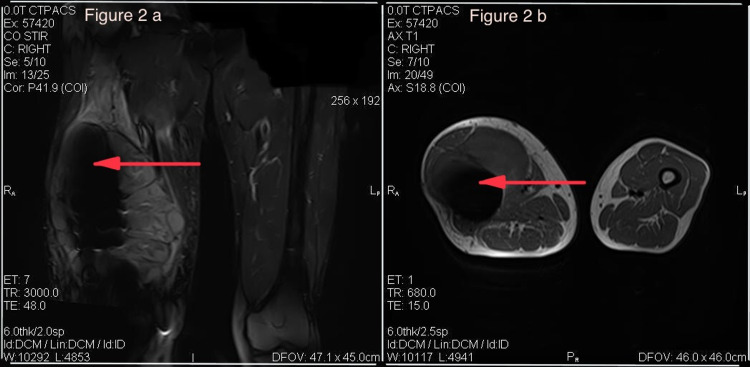
MRI right upper thigh coronal (a) and axial (b) sections showing heterogenous collection wrapping around the bone displacing the vasti muscles and mild edema signal in the adductor magnus, brevis, and longus muscles.

Physical examination revealed a swelling measuring 10 x 15 cm on the anterior lateral aspect of the distal thigh with mild restriction of knee joint movement. The overlying skin was red, warm, and tender to touch without any other significant skin changes. The remainder of the examination was unremarkable. Complete blood count revealed an elevated count of White blood cells (13,900/µL) and platelets (572,000/µL). The right femur x-ray showed dynamic compression plates (DCPs) and surrounding hyperlucency (Figure 1). Ultrasound and MRI of the right lower extremity were obtained, which showed findings consistent with an abscess and the presence of necrotic tissue probably secondary to the DCP placed after the MVC in 2003 (Figures 2a, 2b). A plan was made to drain the abscess and debride necrotic tissue via incision and drainage.

Incision and drainage were abruptly stopped as the initial incision resulted in copious blood loss causing hemodynamic instability. The patient was stabilized with nine units of packed red blood cells, four units of platelets, and four units of fresh frozen plasma along with 1 gram of calcium gluconate. Tissue samples were taken for culture and sensitivity and histopathology. After the unsuccessful procedure, the patient’s pain improved, but the blood count continued to suggest an infective process, so empiric antibiotics were started.

**Figure 3:**
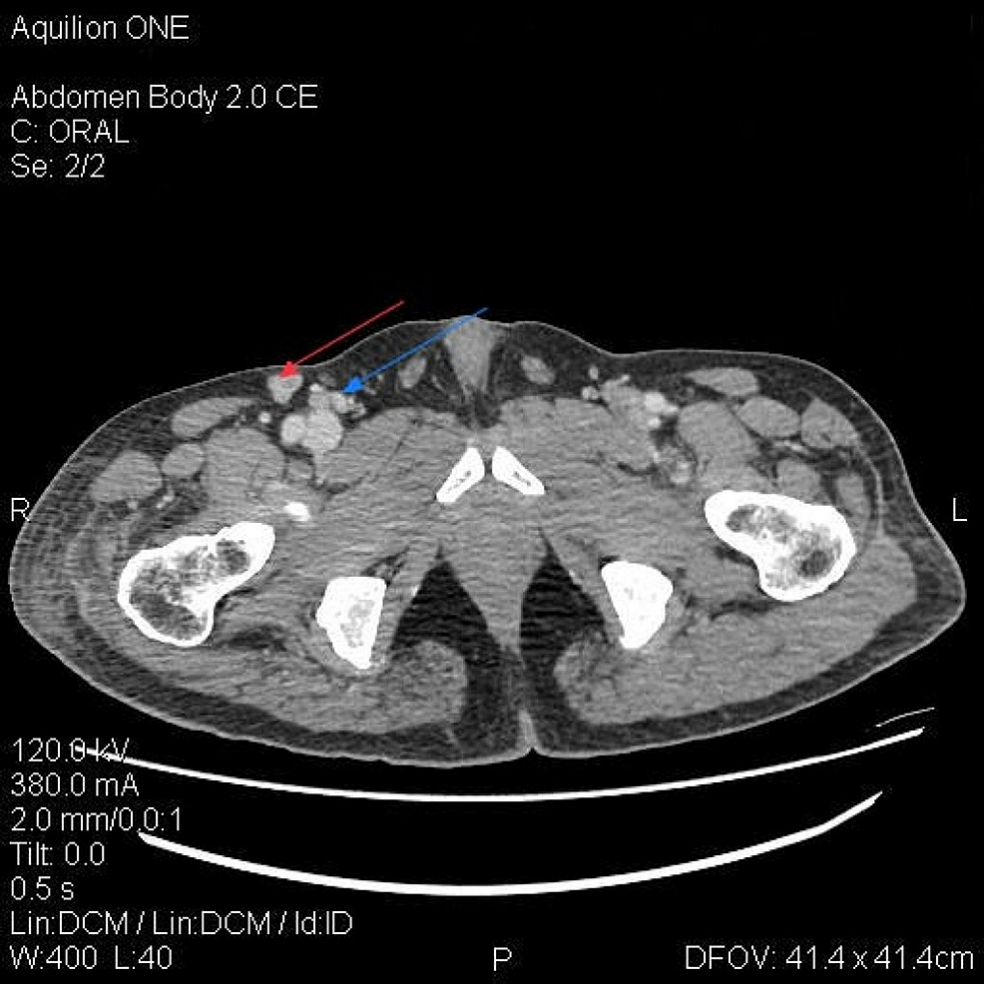
CT pelvis with contrast showing enlarged right inguinal (red arrow) and right external iliac lymph nodes (blue arrow) with mild subcutaneous strandy changes in the right upper thigh.

The histopathology report revealed a tumor comprised of sheets of atypical cells having vesicular nuclei and eosinophilic cytoplasm. At loci, the cells showed prominent plasmacytoid appearance and areas of necrosis. Immunohistochemical staining of the specimen was positive for CD138, MUMI, and CD 56 and negative for CD20 and ALK. CT Pelvis with contrast (Figure 3) revealed enlarged right inguinal, common, internal, and external iliac lymph nodes with mild subcutaneous strandy changes in the visualized right upper thigh. Free Kappa and Lambda chains were also noted to be high (70.3 mg/L and 52.6 mg/L, respectively). Serum LDH was elevated (1,456U/L) while serum protein electrophoresis, free light chain assays, bone marrow biopsy, bone survey, serum calcium levels, iron panel, and peripheral smear results were unremarkable. Hepatitis and HIV profiles (HBsAg, HBV DNA, HCV Ab, HIV Ab) were negative. Thus, a diagnosis of PBL was confirmed. The patient was then placed on ECHOP (etoposide, prednisone, vincristine, cyclophosphamide, and doxorubicin). The patients completed the six cycles of chemotherapy with marked improvement in symptoms. However, the patient was lost to follow up after completing six cycles of chemotherapy due to transportation and financial issues.

## Discussion

PBL is an aggressive form of diffuse large B-cell lymphoma. It is characterized by gradual expression of transcription factors associated with the plasmacytic differentiation, CD38, CD138, MUM1, Blimp1, and XBP1, with decreased expression of CD20 and PAX5 [11]. Although most cases occur in patients with HIV, EBV infection, or are immunocompromised [2,3], approximately 35% of subjects in a recent meta-analysis occurred in immunocompetent individuals [12-14]. The oral cavity is the most common site affected by PBL, followed by the gastrointestinal tract, lymph node, and skin [15]. As with other lymphomas, patients usually present with symptoms that depend on the organ affected, as well as fever, weight loss, and night sweats (B symptoms). Because PBL is very rare, it has not been easy to establish a chemotherapy regimen that would be considered standard treatment. The treatments have included CHOP (cyclophosphamide, doxorubicin, vincristine, and prednisone), ECHOP or CHOP-like regimens, Hyper-CVAD-MA (hyper-fractionated cyclophosphamide, vincristine, doxorubicin, dexamethasone, and high-dose methotrexate and cytarabine), CODOX-M/IVAC (cyclophosphamide, vincristine, doxorubicin, high-dose methotrexate/ifosfamide, etoposide, and high-dose cytarabine), COMB (cyclophosphamide, Oncovin, methyl-CCNU, and bleomycin), and infusional ECHOP (etoposide, prednisone, vincristine, cyclophosphamide, and doxorubicin) [16,17]. The prognosis of patients with PBL is usually poor with a median overall survival between six and 19 months, regardless of HIV status [3,13].

Only two cases of PBL presenting as a soft tissue mass in immunocompetent patients have been published so far [11,12]. Our case is unique as it occurred in thigh muscles, whereas the previous two reports were reported PBL involving the arm and heel. Furthermore, it is the first case to occur at the site of DCP plates insertion. Our patient initially presented with symptoms suggestive of osteomyelitis from possibly infected DCP. The patient's history, clinical presentation, and gross appearance of the lesion were also consistent with this diagnosis. However, a tissue biopsy revealed a diagnosis of PBL. One of the important differential diagnoses in our case was plasmablastic myeloma. The absence of bone marrow involvement or hypercalcemia, renal dysfunction, and positive CD56 in our patient made plasmablastic myeloma an unlikely diagnosis since most experts consider ECHOP as a first-line treatment for PBL [16]. The patient was started on ECHOP for six cycles.

Our case highlights the importance of adequate histopathological evaluation for accurate diagnosis and appropriate management of PBL. Physicians should keep in mind the possibility of this diagnosis in such cases. In summary, our case confirms the paramount role of early clinical suspicion of PBL and correct diagnosis in choosing the most appropriate treatment.

## Conclusions

PBL diagnosis requires a high degree of suspicion and attention from both general physicians and orthopedic surgeons. Because PBL is an aggressive NHL, delayed diagnosis can negatively impact both treatment and survival. Therefore, in particular, orthopedic surgeons should be aware of the varied presentation of this disease, which is often mistaken for soft tissue abscess, osteomyelitis, or an infected implant, leading to a delay in the most appropriate diagnostic evaluation and treatment.
